# Markovian analysis of unreliable multi-machine flexible manufacturing cell

**DOI:** 10.1371/journal.pone.0259247

**Published:** 2022-02-01

**Authors:** Mohammad Hamasha, Sa’d Hamasha, Faisal Aqlan, Osama Almeanazel

**Affiliations:** 1 Department of Industrial Engineering, The Hashemite University, Zarqa, Jordan; 2 Department of Industrial Engineering, Auburn University, Auburn, Alabama, United States of America; 3 J.B. Speed School of Engineering, University of Louisville, Louisville, Kentucky, United States of America; Zhejiang University, CHINA

## Abstract

In this paper, a Markovian model is constructed to test a flexible manufacturing cell’s (FMC) performance. The considered FMC includes a conveyer belt, robot, and n machines. The conveyer belt delivers the working part to the robot, and the robot picks it up and loads it onto the machines. The movement of a working part from one step to the next depends on the availability of the tool in the next step (i.e., conveyer belt, robot, and machine). Any machine is assumed to potentially fail during the processing time as a result of high loading stresses. First, a Markovian model is constructed for single-machine and double-machine FMCs. Then, a generalized FMC with an n-machine is constructed. The introduced model is illustrated with two numerical examples for both the single- and triple-machine. The Markov chain model can be used to estimate the FMC performance measures (i.e., overall utilization of machines and production rate). It is used to analyze the response of these measures under varying parameters (i.e., conveyor belt delivery rate, robot loading rate, processing rate of a machine, failure rate of a machine, and down machines’ repairing rate). Moreover, an economic model based on the Markov chain model is introduced to analyze the FMC’s net profit under these varying parameters.

## 1. Introduction

Flexible manufacturing systems (FMSs) consist of automated machines that perform different operations and produce different products with a material handling system and a centralized computer system to control the system [1, 2]. FMSs have been widely used in today’s industry, especially when there is a wide variety in demand. They can operate a variety of items simultaneously, and they allow variable routes and quick changeovers. The FMS has many advantages over traditional manufacturing systems, such as higher utilization, less floor space, lower flow time and work in process, and lower labor costs. Some of these advantages were discussed by [3–6]. Reddy et al. [4] noted the greater productivity as the main advantage of FMSs. Smith et al. [5] presented a survey of the characteristics of United States FMSs. Moreover, Mansfield [6] wrote about the economic effects of FMSs in different countries.

On the other hand, FMSs possess many disadvantages [3, 7–9], such as technological difficulty in the exact positioning of components and the same necessary time to process a part. Moreover, it is very costly to overcome this difficulty [3]. Therefore, the high investment cost prevents small manufacturing companies from adopting FMS fully. Instead, many adopt flexible manufacturing cells (FMCs). The FMC is a workplace that uses numerically controlled (NC) robots and one or more machines.

In the last few years, many algorithms and models have been developed to handle different issues in the FMCs, especially the routing and scheduling of working parts in different manufacturing stages.

Fahmy et al. [10] proposed mixed-integer programming formulations for the deadlock-free scheduling problem of FMCs. In detail, a heuristic model is proposed, and mathematical experiments are executed to assess the performance (i.e., efficiency and computational time). Tu and Sorgen [11] proposed three basic criteria and a coding system to implement the FMC’s transportation control and scheduling system. Their approach was able to provide the shortest route for the part flow. Azadeh et al. [12] presented a decision-making model for optimization of operator allocation in a flexible manufacturing cellular system using computer simulation (CS) and genetic algorithm (GA). An integrated approach determined the number of cross-trained operators and optimum operators’ layout concerning cellular condition in a FMS. [13–15] are examples of researchers who have designed mathematical models and algorithms for FMC scheduling. [16–18] developed models for dynamic and static scheduling in FMCs.

Some researchers have also modeled the performance of the FMC using different approaches, such as optimization, artificial intelligence, simulation, decision theory, fuzzy logic, stochastic process, etc. Pezzellaa et al. [19] developed a genetic algorithm for flexible job flow scheduling. [20–23] conducted a stochastic analysis to assess the FMC’s performance. However, system characteristics, such as design, process flow, and reliability of FMC, significantly influence the performance. Vineyard and Meredith [24] studied the effects of maintenance policies on failure types of FMS by involving the simulation in real systems. Aldaihani and Savsar [23] presented a Markov chain model for FMC that consists of a pair of machines and a specified handling system. The model was used to determine the performance of a flexible manufacturing cell (FMC) under variable operational conditions, including random machining times, random loading and unloading times, and random pallet transfer times. Aldaihani and Savsar [25] extended their research in 2005 by providing another Markov chain model; this model includes failure and repairing rates of the machines. Moreover, they presented another stochastic model for the FMC that consisted of a pair of machines, robots, and a specified handling system.

[26–29] have used simulation to estimate the performance of the FMC, while [28, 30, 31] have presented Markov chain models to evaluate the performance of the FMC. In addition, Koulamas [29] introduced a semi-Markov model to investigate the influence of tool failures on the FMC’s performance.

In general, majority of the researchers use either the Markov chain (e.g., this paper) or a simulation to handle FMC. The Markov chain leads to a very accurate result while the simulation can handle a wide range of probability distribution. In the present work, we extended the Hamasha et al. [32] model of the FMC with a Markovian nature. We used the model’s general framework and notations. However, we added details and realistic considerations aside from the reliability. Furthermore, we provided an economic model to optimize the profit, which is an extra component from their model. In the current research, the introduced Markov chain model can determine the performance of FMC. The model gives an exact estimation of the overall machine(s) utilization (OAMU) and the production rate (PR). In addition, the failure rate of machines and the machines’ repairing rate are considered. A model of the FMC with a single machine is introduced first. Then, the model is upgraded to deal with a double machine and generalized to handle multiple machines. Finally, numerical solutions and economic analysis FMCs are conducted to optimize the different parameters and indicate their impact on the economy.

## 2. System description

**Fig 1:**
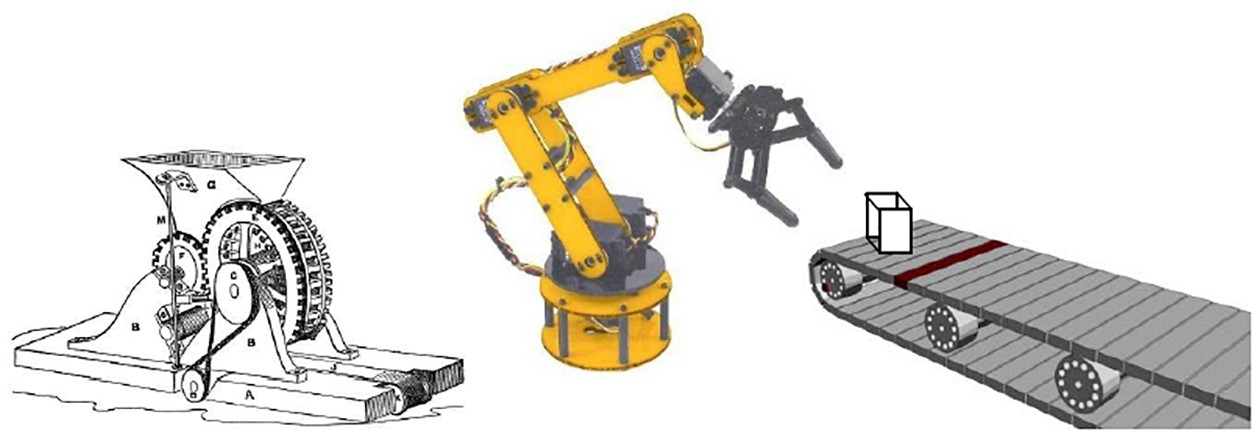
Schematic illustration of flexible manufacturing cell with single machine.

This paper is based on a real case and realistic assumptions, as explained in Hamasha et al. [32]. Further, we introduced the status of failure in the current extension to better align it with reality. The FMC consists of n identical machines, one automated handling device (robot), and one conveyor belt. A system with one machine configuration appears below in Fig 1. The conveyor belt delivers a part to the robot. The robot works as a pick up and place device. It picks up the part that is transported via the conveyor belt and places it in one of the available machines. The machines will then process the working part and push it to the next manufacturing process without involving the robot in the unloading process. The delivery of a working part from one step to the next depends on the availability of the tool in the next step (i.e., conveyer belt, robot, and machine). The machines are subject to failure during processing as a result of high loading stresses. However, it is assumed that machine failure does not occur during idle time. This is because the idle machine stays at rest—and may even be completely turned off—before receiving the next part; it then starts to work again. Furthermore, if any machine fails, the entire system stops working until the appropriate maintenance procedures are completed. This assumption is adopted because the workers cannot work on the failed machine while the unit (FMC) is operating. This is a serious cause for concern with regard to safety issues.

The proposed FMC model has the following characteristics and represents the general case for FMCs in the industry: the conveyor belt delivers a single part; the robot transfers a single part to an available machine; each machine processes a single part; only one part is accepted as in-process inventory for conveyor belt-robot and robot-machines; machines are similar; all machines have a similar loading rate; failure and repairing rates are the same for all machines; and failure can occur only if the machine is processing the uploaded part.

Many parameters affect the performance of the FMC, such as conveyor belt delivery rate, loading rate of the robot, processing rate of the machine, machine failure rate, and maintenance rate of down machines. In addition, the loading time of the robot, processing time of the machine, conveyor belt delivery time, time to failure, and maintenance time of the down machines are assumed to be exponentially distributed.

## 3. Proposed model and methodology

**Fig 2:**
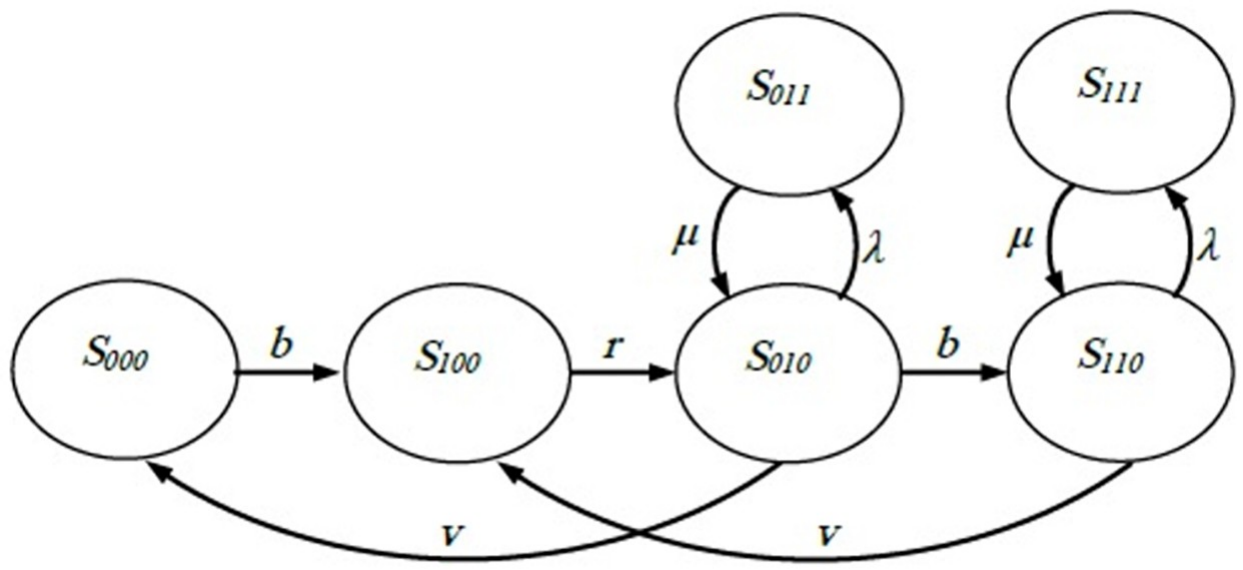
State transition diagram of single-machine FMC.

The model’s symbols are explained in S1 Appendix. The standard Markov chain has two types of components: 1) the probability at each state that is dimensionless, and 2) transition rate from one state to another. All transition rates in a system should be per the same unit. Three variables are used to describe the state of the system: the part availability to be grasped by the robot (i), the number of the parts that are processed by the machines (j), and the status of the machines (k). In case of FMCs with one machine, each one of the three variables can take the value of either 0 or 1, so the system should be in one of the following states: (S_000_), (S_100_), (S_010_), (S_110_), (S_011_) and (S_111_). Fig 2 presents the transition flow between the different states in a single machine FMC. The system can be in state (S_000_) if no part is available to pick up, no part is in the machines, and the machine has not failed. However, if one part comes with the conveyor belt, the robot becomes available for pick up and the system is subsequently transferred to state (S_100_) with a conveyer belt delivery rate (b). Once the robot loads the machine with a part, the system is transferred to state (S_010_) with a state transition rate (i.e., loading rate) of the robot (r). At state (S_010_), the system can move to state (S_110_), (S_000_), or (S_011_). Particularly, it moves forward to state (S_110_) with a rate of (b) if a new part arrives and becomes available for pick up by the robot. Moreover, it moves back to the state (S_000_) with a machine processing rate (v) when the machine finishes processing the working part, or it moves to the state (S_011_) with a failure rate (λ) when the machine fails during the processing. At state (S_110_), there is a chance of moving to state (S_100_) with a rate of (v) if the machine finishes working on the uploaded part, or it may move to state (S_111_) with a rate of (λ) if the machine is down. If the machine is down (states S_011_ and S_111_), the system returns to the original state (the state before the occurrence of the failure) with a rate of (μ) if the appropriate maintenance takes place. It is evident that the system is recurrent.

Under the steady-state condition, the probability to transit from outside the state is equal to the probability to transit from the state. In other words, the net flow rate is zero at each state (the flow-in equals flow-out). The following equations are extracted for the stochastic FMC with a one-machine system.


$$\begin{array}{l} {\text{vP}}_{010}-{\text{bP}}_{000}=0\\ {\text{bP}}_{000}+{\text{vP}}_{110}-{\text{rP}}_{100}=0\\ {\text{rP}}_{100}+{\mu \text{P}}_{011}-\left(\lambda +\text{b}+\text{v}\right){\text{P}}_{010}=0\\ {\text{bP}}_{010}+{\mu \text{P}}_{111}-\left(\lambda +\text{v}\right){\text{P}}_{110}=0\\ {\lambda \text{P}}_{010}-{\mu \text{P}}_{011}=0\\ {\lambda \text{P}}_{110}-{\mu \text{P}}_{111}=0 \end{array}$$ (1)

Furthermore, the total sum of state probabilities is 1, as shown in the following equation.


$${\text{P}}_{000}+{\text{P}}_{100}+{\text{P}}_{010}+{\text{P}}_{110}+{\text{P}}_{011}+{\text{P}}_{111}=1$$ (2)

Assume all rates (i.e., b, r, v, λ, and μ) are known. Eqs (1) and (2) consist of seven equations with six unknowns. The set is solvable to estimate the probability of each state or, in other words, the percent of time that the system stays in each state. The OAMU and the PR can be estimated as seen in the following two equations.

$$\text{OAMU}=\left({\text{P}}_{010}+{\text{P}}_{110}\right)\times 100\text{\%}$$ (3)

$$\text{PR}=\left({\text{P}}_{010}+{\text{P}}_{110}\right)\times \text{v}$$ (4)

where (P_010_ + P_110_) × 100% is the percent of the time that the machine is busy.

**Fig 3:**
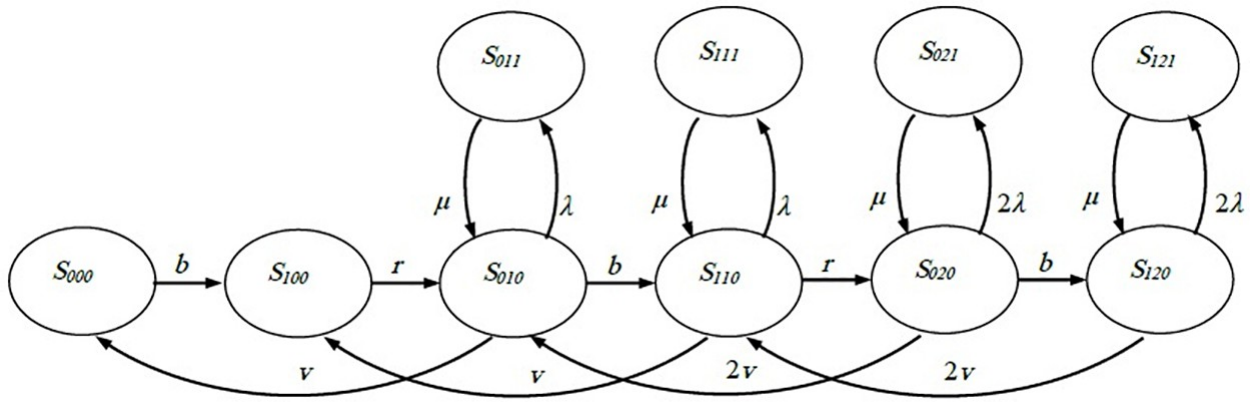
State transition diagram of double-machine FMC.

The double-machine FMC system can be in one of the following states: (S_000_), (S_100_), (S_010_), (S_110_), (S_020_), (S_120_), (S_011_), (S_111_), (S_021_), and (S_121_). Fig 3 presents the transition flow between the different states. If no part is available for pickup by the robot and both machines are idle, the system is in the state (S_000_). However, the system is transferred to state (S_100_) with a rate of (b) if a part is brought by the conveyer belt and becomes available for pickup by the robot. Once the robot feeds the held part to a machine, the system status is changed to (S_010_). The rate of this transition is (r). State (S_010_) can transit to state (S_110_) with a rate of (b) if a new part comes to the robot, or it can transit to state (S_000_) with a rate of (v) if the machine finishes processing the part. Moreover, if the processing machine fails, it can transit to state (S011) with a rate of (λ). State (S_110_) can transit to state (S_020_) with a rate of (r) if the robot loads the new part to the second machine, or it can transit to state (S_010_) with a rate of (v) if the machine finishes processing the part. Furthermore, if a machine fails during processing, it can transit to state (S111) with a rate of (λ). State (S_020_) can transit to state (S_120_) with a rate of (b) if a new part comes to the robot, or it can transit to state (S_100_) with a rate of (2v) if any machine finishes processing the part. In addition, it can transit to state (S_021_) with a rate of (2λ) if any machine fails during processing. State (S_120_) can only transit to either state (S_110_) or state (S_121_). It can transit to state (S_110_) with a rate of (2v) if any machine finishes processing a part, or it can transit to state (S_121_) with a rate of (2λ) if any machine fails during processing. Once a processing machine fails (states S_010_, S_110_, S_020_, and S_120_), the k is changed from 0 to 1. For example, if a processing machine fails at state (S_120_), the state is changed to (S_121_). However, if the down machine is fixed, the value of k is changed from 1 to 0 again. This transition happens at a rate of (μ). In other words, if the down machine at state (S_121_) is fixed, the state is changed to (S_120_).

Similarly, the net flow rate (the difference between the rates of flow-in and flow-out) at each state is equal to zero. Therefore, the following equations are extracted from the double-machine FMC transition diagram.


$$\begin{array}{l} {\text{vP}}_{010}-{\text{bP}}_{000}=0\\ {\text{bP}}_{000}+{\text{vP}}_{110}-{\text{rP}}_{100}=0\\ {\text{rP}}_{100}+{\text{vP}}_{020}+{\mu \text{P}}_{011}-\left(\lambda +\text{v}+\text{b}\right){\text{P}}_{010}=0\\ {\text{bP}}_{010}+{\text{vP}}_{120}+{\mu \text{P}}_{111}-\left(\lambda +\text{v}+\text{r}\right){\text{P}}_{110}=0\\ {\text{rP}}_{110}+{\mu \text{P}}_{021}-\left(2\lambda +2\text{v}+\text{b}\right){\text{P}}_{020}=0\\ {\text{bP}}_{020}+{\mu \text{P}}_{121}-\left(2\lambda +2\text{v}\right){\text{P}}_{120}=0\\ {\lambda \text{P}}_{010}-{\mu \text{P}}_{011}=0\\ {\lambda \text{P}}_{110}-{\mu \text{P}}_{111}=0\\ 2{\lambda \text{P}}_{020}-{\mu \text{P}}_{021}=0\\ 2{\lambda \text{P}}_{120}-{\mu \text{P}}_{121}=0 \end{array}$$ (5)

The total sum of state probabilities is 1, as shown in the following equation.


$${\text{P}}_{000}+{\text{P}}_{100}+{\text{P}}_{010}+{\text{P}}_{110}+{\text{P}}_{020}+{\text{P}}_{120}+{\text{P}}_{011}+{\text{P}}_{111}+{\text{P}}_{021}+{\text{P}}_{121}=1$$ (6)

Similarly, assume the system rates (i.e., b, r, v, λ, and μ) are known. Eqs (5) and (6) consist of eleven equations with ten unknowns. The set is solvable to estimate the percent of the time the system is in each state (the probability of each state). The OAMU is estimated by adding the percent of the time that the two machines are processing (P_020_ + P_021_), in addition to half of the time that one machine (out of the two) is processing (P_010_ + P_011_), as only half of the system is producing. The OAMU is addressed in Eq 7. The PR is estimated by multiplying the OAMU with the maximum operational production rate (2v), as expressed in Eq 8.


$$\text{OAMU}=\left[\frac{1}{2}\left({\text{P}}_{010}+{\text{P}}_{110}\right)+\left({\text{P}}_{020}+{\text{P}}_{120}\right)\right]\times 100\text{\%}$$ (7)

$$\text{PR}=\left[\frac{1}{2}\left({\text{P}}_{010}+{\text{P}}_{110}\right)+\left({\text{P}}_{020}+{\text{P}}_{120}\right)\right]\times 2\times \text{v}$$ (8)

**Fig 4:**
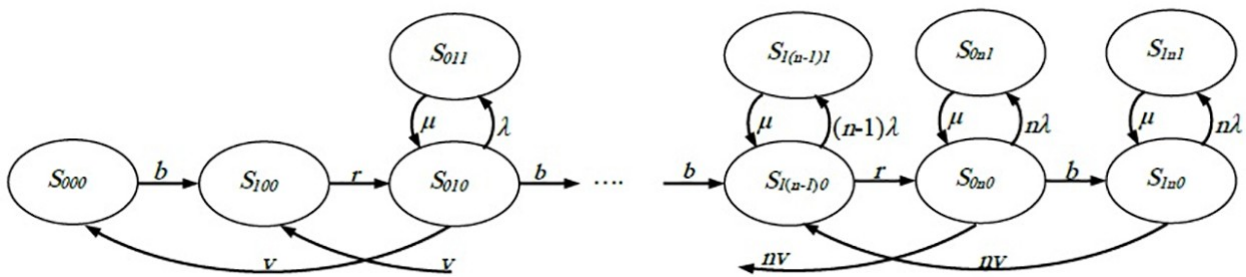
State transition diagram of n-machine FMC.

Several observations in single- and double-machine FMC models can be highlighted to help build a general model (i.e., n-machine FMC) as follows. 1) The number of possible states is 4n + 2. For example, there are ten states in the double-machine systems (six states when all machines are working and four states when a machine is down). 2) The states when the machines are working can be arranged in a line as follows: (S_000_, S_100_, S_010_, S_110_, S_020_, S_120_, S_030_,…, S_1(n-1)0_, S_0n0_, S_1n0_). It can be noticed that i changes alternatively from 0 to 1 and then from 1 to 0, j increases by one every two steps (i.e., 0, 0, 1, 1,…, n-1, n-1, n, n), and k equals 0 at all transitions. 3) The transition rate can be depicted in regular form (i.e., it can transfer to the next state with a rate of (b) if the robot stage is empty and (r) if the robot stage is occupied, so the rate changes with the state’s series alternatively between b and r). 4) The state can go two steps back with a rate of jv. However, there is no back transition when j = 0 (i.e., S_000_ and S_100_). 5) At each state of the series—with the exception of the first two states—the state can transit out of this series if a machine fails. At this transition, k changes from 0 to 1 with a transition rate of jλ. However, the transition rate from the down machine’s state back to the series (when the maintenance took place) is μ. Therefore, the system with n machines can be expressed in the transition diagram, as shown in Fig 4.

The following system of 4n + 2 equations can be extracted from the above transition flow diagram depending on the fact that the sum of transition flow at each state is equal to 0.


$$\begin{array}{l} {\text{vP}}_{010}-{\text{bP}}_{000}=0\\ {\text{bP}}_{000}+{\text{vP}}_{110}-{\text{rP}}_{100}=0\\ {\text{rP}}_{100}+2{\text{vP}}_{020}+{\mu \text{P}}_{011}-\left(\lambda +\text{v}+\text{b}\right){\text{P}}_{010}=0\\ {\text{bP}}_{010}+2{\text{vP}}_{120}+{\mu \text{P}}_{111}-\left(\lambda +\text{v}+\text{r}\right){\text{P}}_{110}=0\\ {\text{rP}}_{110}+3{\text{vP}}_{030}+{\mu \text{P}}_{021}-\left(2\lambda +2\text{v}+\text{b}\right){\text{P}}_{020}=0\\ {\text{bP}}_{020}+3{\text{vP}}_{130}+{\mu \text{P}}_{121}-\left(2\lambda +2\text{v}+\text{r}\right){\text{P}}_{120}=0\\ .\;\;\;\;\;\;\;.\;\;\;\;\;\;\;.\\ .\;\;\;\;\;\;\;.\;\;\;\;\;\;\;.\\ .\;\;\;\;\;\;\;.\;\;\;\;\;\;\;.\\ {\text{rP}}_{1\left(\text{n}-1\right)0}+{\text{nvP}}_{0\text{n}0}+{\mu \text{P}}_{0\left(\text{n}-1\right)1}-\left(\left(\text{n}-1\right)\lambda +\left(\text{n}-1\right)\text{v}+\text{b}\right){\text{P}}_{0\left(\text{n}-1\right)0}=0\\ {\text{bP}}_{0\left(\text{n}-1\right)0}+{\text{nvP}}_{1\text{n}0}+{\mu \text{P}}_{1\left(\text{n}-1\right)1}-\left(\left(\text{n}-1\right)\lambda +\left(\text{n}-1\right)\text{v}+\text{r}\right){\text{P}}_{1\left(\text{n}-1\right)0}=0\\ {\text{rP}}_{1\left(\text{n}-1\right)0}+{\mu \text{P}}_{0\text{n}1}-\left(\text{n}\lambda +\text{nv}+\text{b}\right){\text{P}}_{0\text{n}0}=0\\ {\text{bP}}_{0\text{n}0}+{\mu \text{P}}_{1\text{n}1}-\left(\text{n}\lambda +\text{nv}\right){\text{P}}_{1\text{n}0}=0\\ {\lambda \text{P}}_{010}-{\mu \text{P}}_{011}=0\\ {\lambda \text{P}}_{110}-{\mu \text{P}}_{111}=0\\ 2{\lambda \text{P}}_{020}-{\mu \text{P}}_{021}=0\\ 2{\lambda \text{P}}_{120}-{\mu \text{P}}_{121}=0\\ .\;\;\;\;\;\;\;\;.\;\;\;\;\;.\\ .\;\;\;\;\;\;\;\;.\;\;\;\;\;.\\ .\;\;\;\;\;\;\;\;.\;\;\;\;\;.\\ {\text{n}\lambda \text{P}}_{0\text{n}0}-{\mu \text{P}}_{0\text{n}1}=0\\ {\text{n}\lambda \text{P}}_{1\text{n}0}-{\mu \text{P}}_{1\text{n}1}=0 \end{array}$$ (9)

The total sum of state probabilities equals 1, as shown in the following equation.


$$\sum_{\text{i}=0}^{1}\sum_{\text{j}=1}^{\text{n}}\sum_{\text{k}=0}^{1}{\text{P}}_{\text{ijk}}=1$$ (10)

Similarly, suppose the system rates (i.e., b, r, v, λ, and μ) are known. In that case, Eqs (9) and (10) consist of 4n + 3 solvable equations with 4n + 2 unknown state probabilities to find out all steady-state probabilities. The OAMU can be estimated by multiplying the percentage of the busy machines by the percent of the time that these machines are busy. For example, if three machines out of five are working for half of the time, the OAMU equals ⅗ multiplied by ½ (three-fifths of the available machines work for half of the time). In order to calculate this quantity, each percent of the time at each number of busy machines (P_0j0_ + P_1j0_) is multiplied by the percent of busy machines (j/n). The summation is achieved for the number of all busy machines (j). The OAMU is expressed in Eq 11. Moreover, the PR can be calculated by multiplying the OAMU (without 100%) with the maximum production rate (nv), as seen in Eq 12.


$$\text{OAMU}=\sum_{\text{j}=1}^{\text{n}}\left(\frac{\text{j}}{\text{n}}\times ({\text{P}}_{0\text{j}0}+{\text{P}}_{1\text{j}0})\right)\times 100\text{\%}$$ (11)

$$\text{PR}=\sum_{\text{j}=1}^{\text{n}}\left(\frac{\text{j}}{\text{n}}\times ({\text{P}}_{0\text{j}0}+{\text{P}}_{1\text{j}0})\right)\times \text{nv}$$ (12)

## 4. Case problem, result and discussion

In this section, two numerical examples are presented and discussed to demonstrate the models constructed in Section 3. The first example is about the single-machine FMC. The second example, regarding three-machine FMCs, serves as an example of the multi-machine FMC.

### 4.1 Single-machine fmc numerical example

Considering the FMC with the following parameters and rates: one machine, (b = 2 parts/min), (r = 4 parts/min), (v = 3 parts/min), (λ = 0.001389 failures/min) and (μ = 0.0333 machines/min). By using Eqs 1 and 2, the probability of each state is estimated as follows (P_000_ = 0.334, P_100_ = 0.279, P_010_ = 0.223, P_110_ = 0.149, P_011_ = 0.009 and P_111_ = 0.006). Furthermore, the OAMU and the PR are 37.15% and 1.11 parts/min, respectively, according to Eqs 3 and 4.

**Fig 5:**
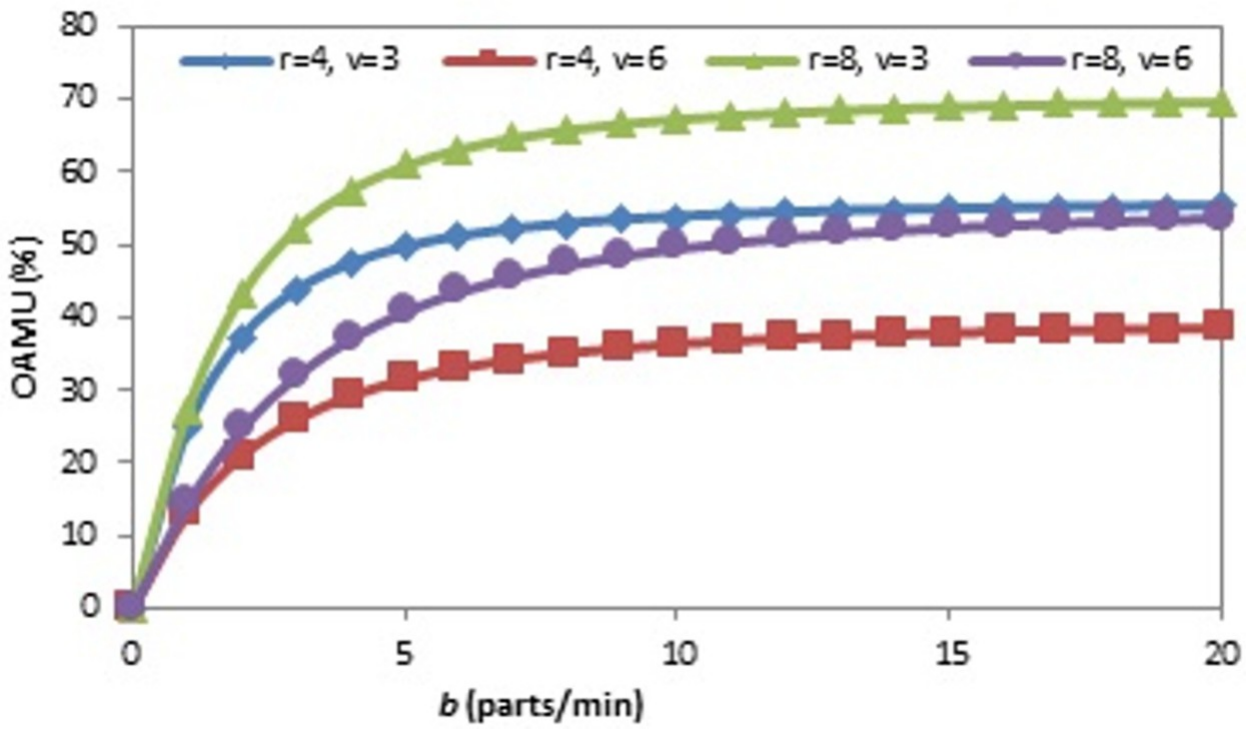
OAMU-b diagram for single-machine FMC.

The relationships between each system parameter and both the OAMU and the PR are drawn by varying one parameter at constant values of the other two and then noticing the response of the OAMU or the PR. The effect of (b) on the OAMU at four different combinations of (r) and (v) is shown in Fig 5. The OAMU increases with (b) at all combination rates to enhance the feeding of parts to the system. However, the OAMU-b curves are concave, which means that the increase of the OAMU is high at a lower value of (b) and low at higher values of (b). At high (b) values, the time to bring a new part with the conveyor belt is negligible compared to the robot loading and machine processing times. Assuming the robot is very fast and the machine processing time is one minute, the OAMU increases significantly if (b) rises from 0.5 to 1 (parts/minute). Conveyor belt delivery time at (b = 0.5) is twice as much as the machine processing time, which means the machine must wait for a new part for at least the same amount of time as the processing time. However, at (b = 1), the conveyer belt delivery time is equal to the processing time, which means the machine may not wait for a new part to come in. This means the enhancement of the OAMU may potentially reach up to 50%. However, if (b) rises from 50 to 50.5 parts per minute (the same increment with the previous case, or 0.5 parts/min), the machine is very busy at both (b) values (the idle time is close to zero). Therefore, the OAMU cannot increase significantly. On the other hand, assuming the robot is not faster than the machine, the robot will hold a higher level of control over the parts being fed to the machine. In this case, the OAMU changes significantly with (b) if the conveyor belt is slower than the robot, and this is because the conveyor belt controls the robot to a large extent. However, if (b) is very high, the parts are usually available for pick up by the robot, and the change of (b) will not have a significant impact on the OAMU.

**Fig 6:**
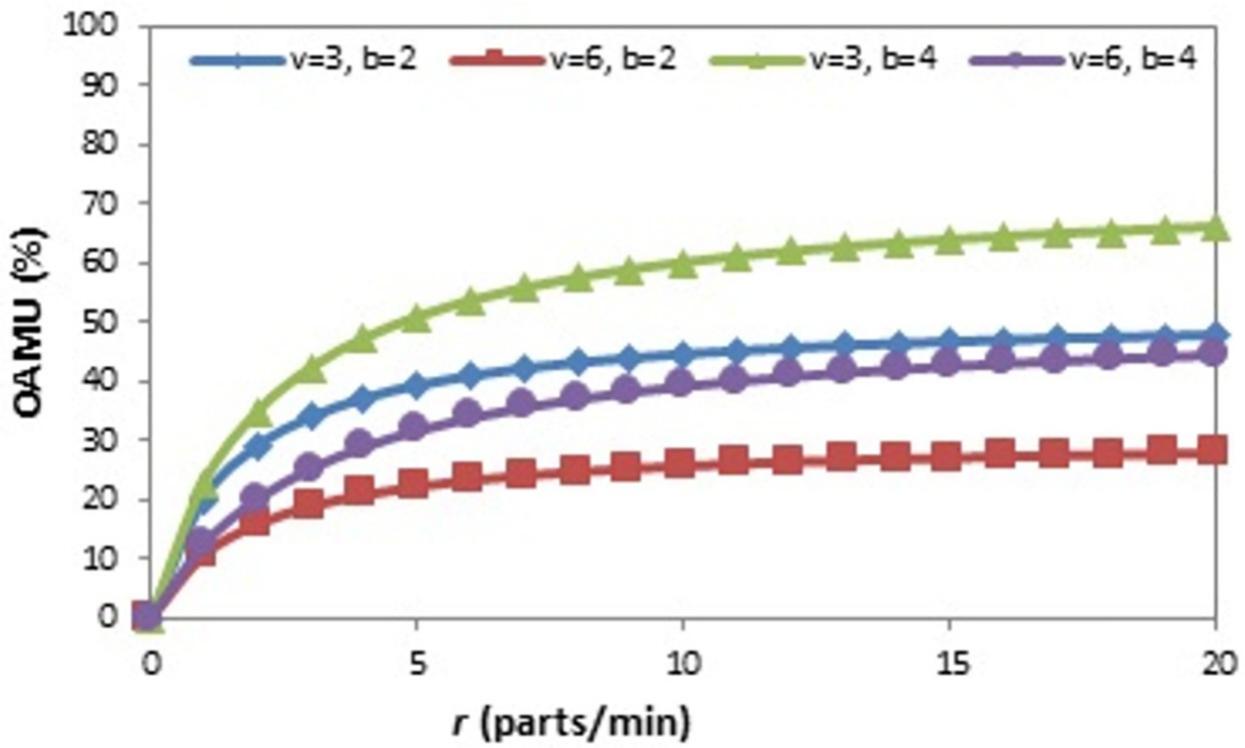
OAMU-r diagram for single-machine FMC.

**Fig 7:**
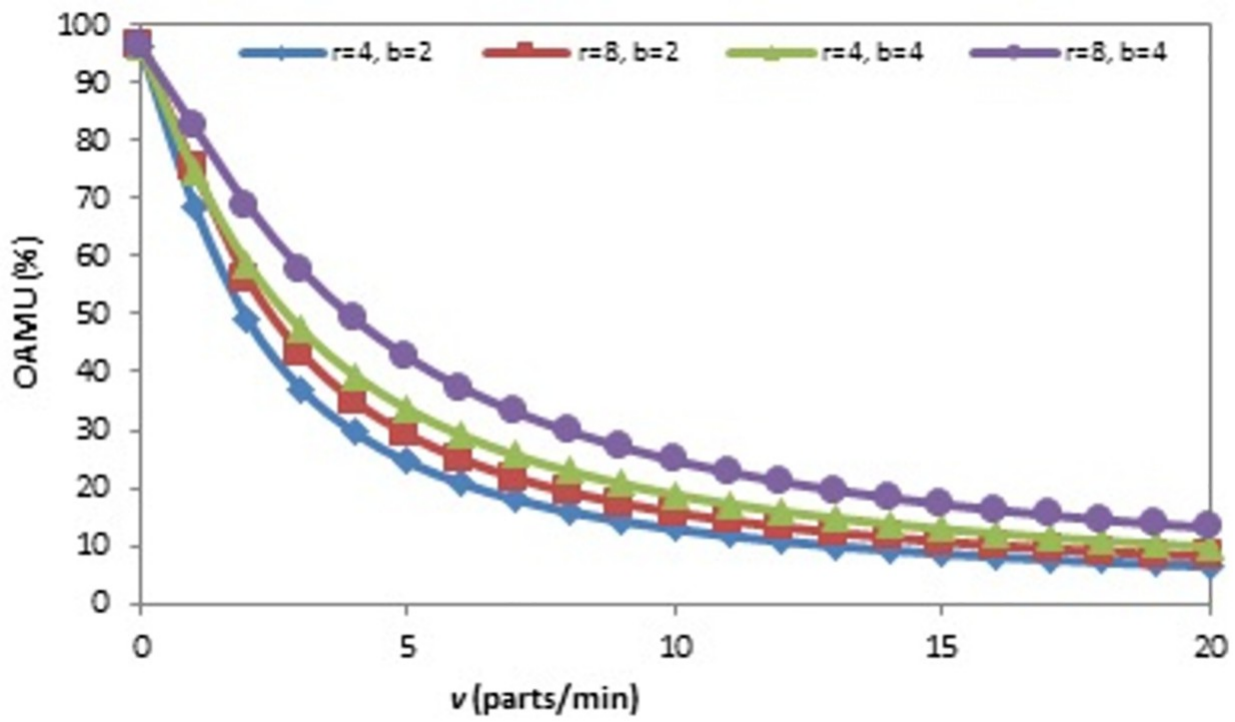
OAMU-v diagram for single-machine FMC.

The effect of the (r) on the OAMU at four different combinations of (b) and (v) is shown in Fig 6. Generally, the increase in (r) directly increases the OAMU. However, the OAMU-v curves are concave, which means that the rate at which the OAMU increases decreases with (r). Theoretically, the OAMU reaches a constant value at infinity of r. Therefore, the robot has a high level of control over the feeding of parts to the machine when the machine is faster than the robot. However, the parts are largely available to be fed to the machine when the (r) is higher than the (v), which leads to less control of the part feeding and, ultimately, less effect on the OAMU. The effect of (v) on the OAMU at four different combinations of (b) and (r) is shown in Fig 7. At all combinations, the OAMU decreases with (v).

**Fig 8:**
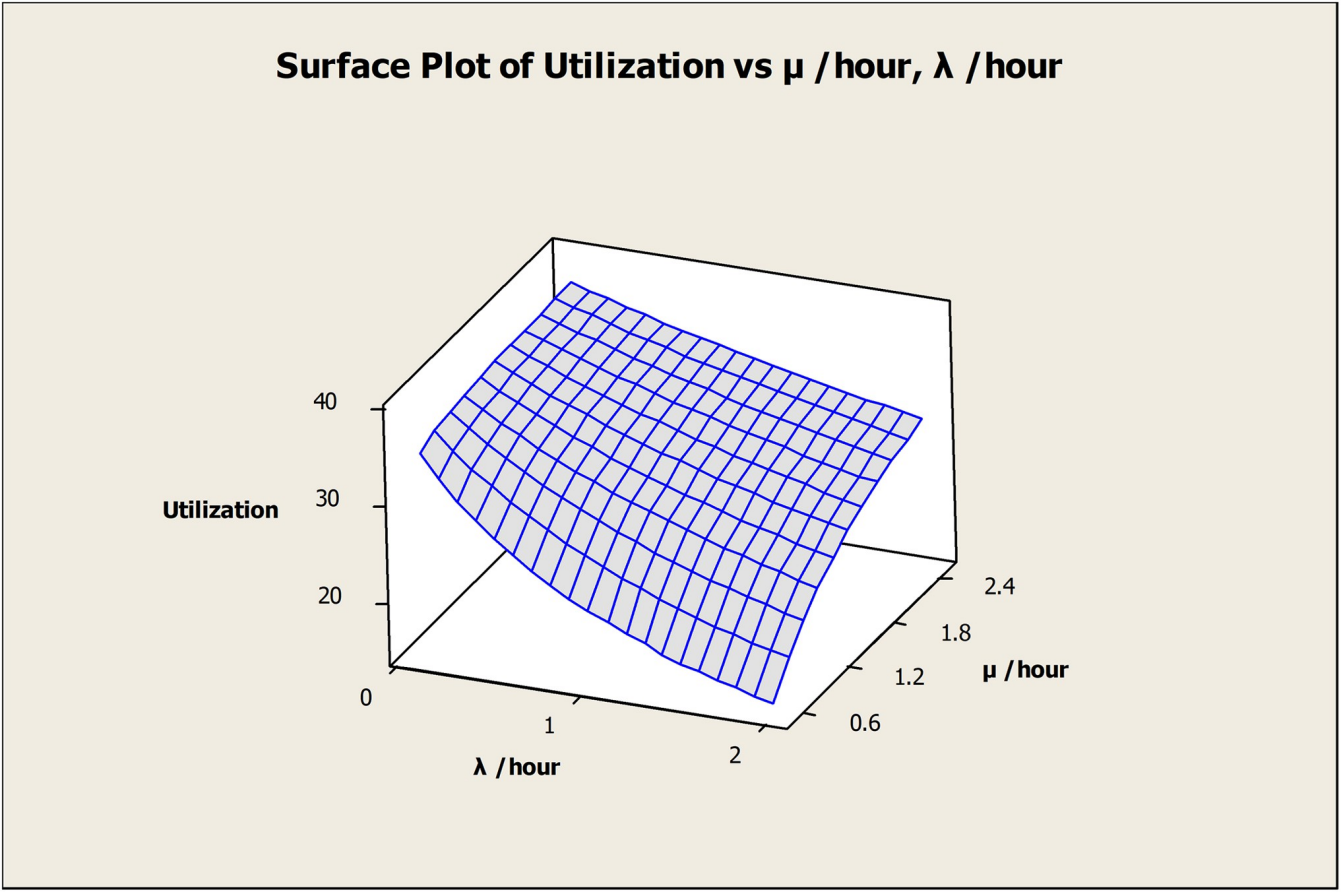
Surface plot of utilization vs μ /hour, λ /hour.

The effect of (λ) and (μ) on the OAMU is shown in Fig 8. Higher (λ) decreases the OAMU due to the increase in the downtime, whereas (μ) increases the OAMU due to the decrease in the downtime. Although the target for any industry is to reduce (λ) and increase (μ) as much as possible, the reliability of the machines and the maintenance cost play significant roles in controlling these rates. Moreover, it is important to note that the OAMU tends to stabilize at higher values of (λ) or (μ). In other words, the OAMU is not sensitive to (λ) at higher (μ) or to (μ) at higher (λ). Usually, (λ) cannot be reduced unless a significant change is performed on the facility (e.g., a change of the current machines or tools). Therefore, optimizing the (μ) by having appropriate levels of maintenance staff is required to reduce the cost.

**Fig 9:**
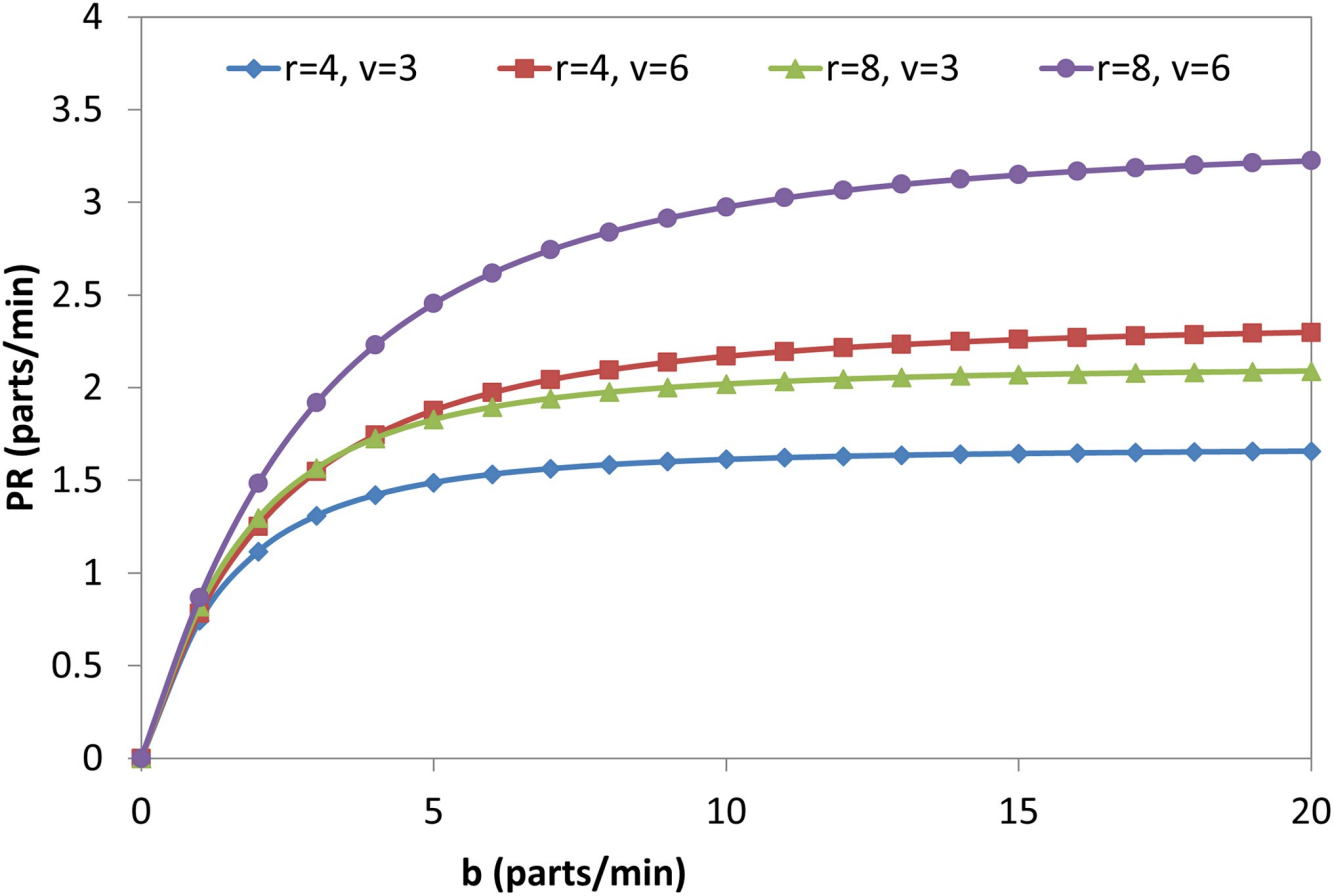
PR-b diagram for single-machine FMC.

**Fig 10:**
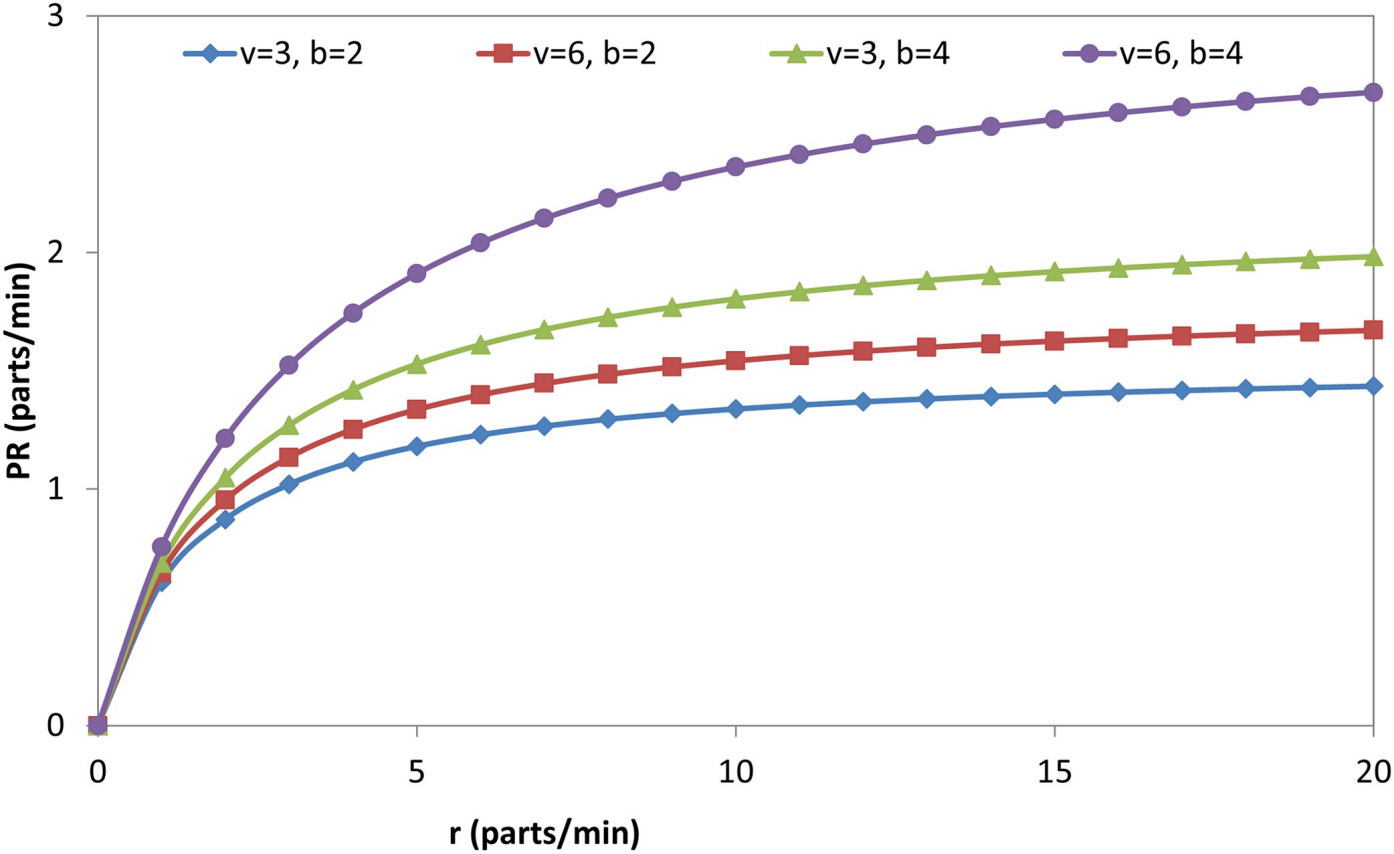
PR-r diagram for single-machine FMC.

**Fig 11:**
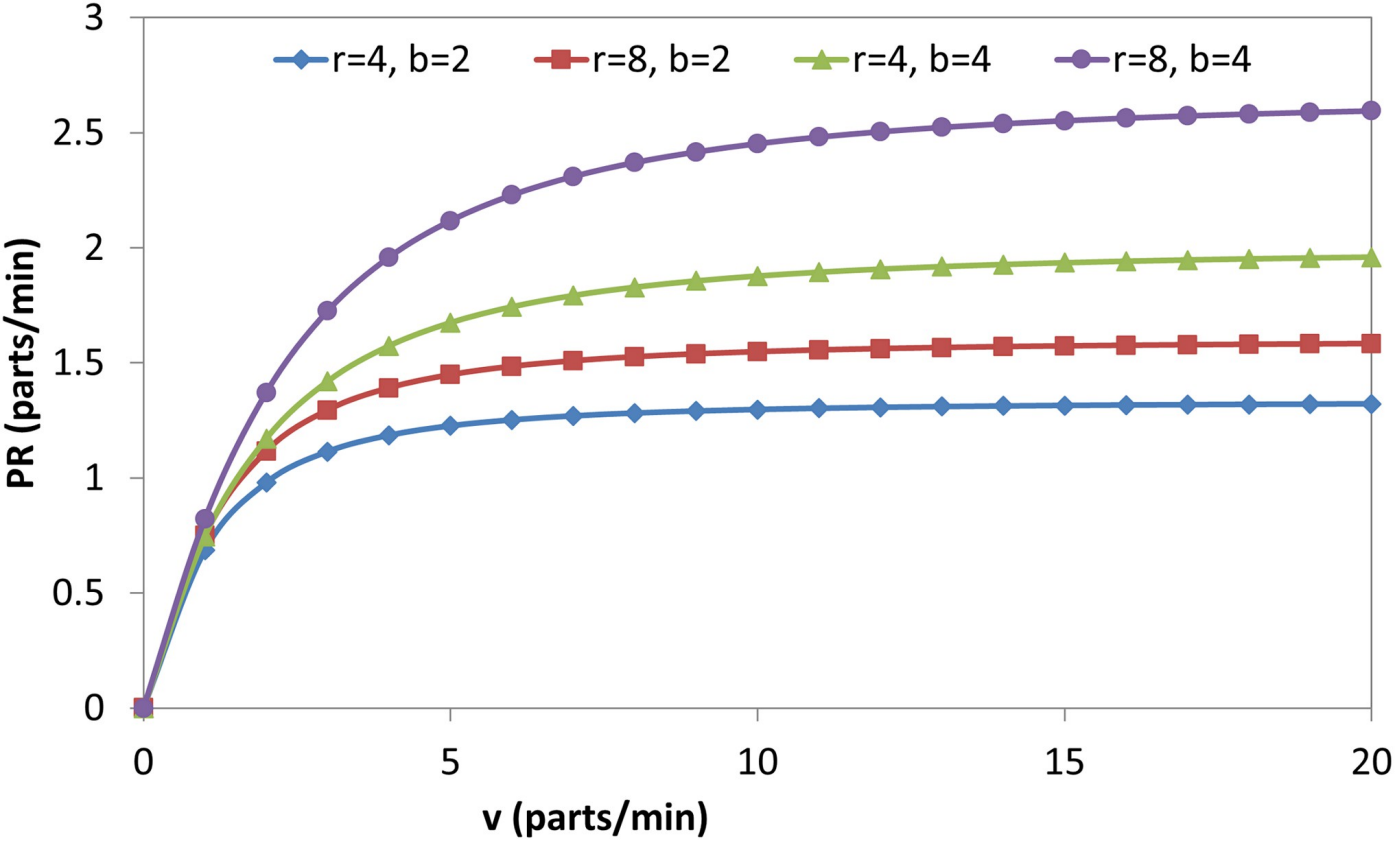
PR-v diagram for single-machine FMC.

**Fig 12:**
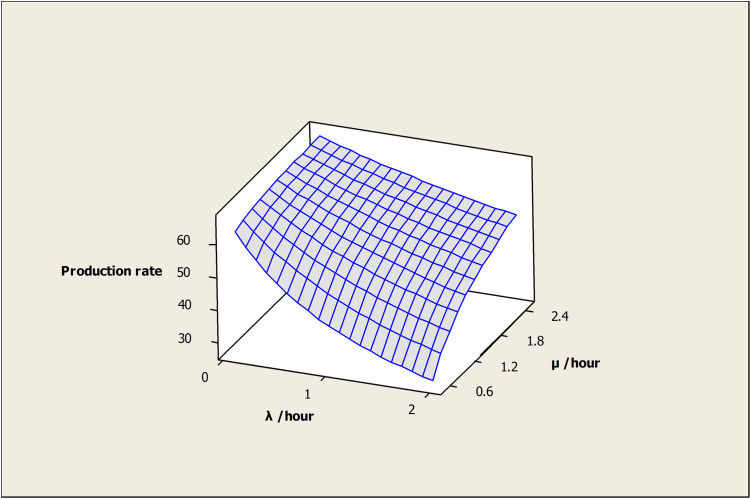
Surface plot of production rate versus μ /hour, λ /hour.

Figs 9, 10, 11 and 12 explain the relationship between the considered parameters and the PR for the FMC with a single machine. Fig 9 shows the relationship between (b) and the PR at four different combinations of (r) and (v). The PR increases with (b), but the rate at which the PR increases decreases with (b) (i.e., PR-b curves are concave). The OAMU equation (Eq 3) shares the PR equation (Eq 4) in the (P_010_ + P_110_) term. The other terms in the equation (i.e., 100% in the OAMU equation and v in the PR equation) are constant for this curve. Therefore, the OAMU-b curves have the same trend as PR-b curves. Fig 10 shows the effect of (r) on the PR at four different combinations of (b) and (v). In general, the PR does increase with (r). However, the PR increase is high at the low (r) level and low at the high (r) level. Since (v) is constant in Fig 10, the OAMU-r curves have the same trend as the PR-r curves. The explanation of the effect of (b) and (r) on the PR is very similar to their effects on the OAMU since the PR follows the OAMU under constant (v). However, if (v) is variable (as in Fig 11), the PR is controlled by a variable quantity (v) that does not exist in the OAMU equation. Therefore, both the PR and the OAMU have different trends with each other. Fig 11 shows the relationship between v and the PR at four combinations of (b) and (r). Generally, the PR increases with the increase of (v). However, the rate of the PR increase decreases with (v). This is because the idle time for the machine is very high under high (v) values, and there is no significant increase in the feeding of parts to the machine with increasing (v) values.

The effect of (λ) and (μ) on the PR is shown in Fig 12. Higher (λ) decreases the PR due to increased downtime, whereas (μ) increases the OAMU due to reduced downtime. It is important to note that the PR tends to stabilize at higher (λ) or (μ) values. In other words, the OAMU is not sensitive to (λ) at higher (μ), and it is not sensitive to (μ) at higher (λ). Usually, (λ) cannot be reduced unless a significant change is performed in the facility (e.g., change of the current machine). Therefore, optimizing the (μ) by having appropriate levels of maintenance staff and technology is required to reduce the cost of production.

### 4.2 Multi-machine fmc numerical example

Consider a triple-machine FMC with the following parameters: (n = 3), (b = 2 parts/min), (r = 5 parts/min), (v = 2 parts/min), (λ = 0.0017 failures/min), and (μ = 0.042 machines/min). Solving the system of Eqs 9 and 10 with the help of MATLAB (version 9) leads to an estimate of the probability of each state as follows: (P_000_ = 0.22, P_100_ = 0.235, P_010_ = 0.22, P_110_ = 0.145, P_020_ = 0.092, P_120_ = 0.036, P_030_ = 0.016, P_130_ = 0.006, P_011_ = 0.009, P_111_ = 0.006, P_021_ = 0.007, P_121_ = 0.003, P_031_ = 0.002 and P_131_ = 0.001). Furthermore, the OAMU and the PR are 23.00% and 1.38 parts/min, respectively, according to Eqs 11 and 12. The computer configuration used in this calculation is as follows: Intel Core i5 processor, Windows 10 Professional x64 Operating System, and 8 GB RAM memory.

The performance of the triple-machine FMC was assessed with the OAMU and the PR. In this case, the OAMU is calculated by adding up the percentage of time that all three machines are utilized, two-thirds of the percentage of time that two machines are utilized (one machine is idle), and one-third of the percentage of time that one machine is utilized (two machines are idle). Similar to the single-machine FMC, the triple-machine FMC numerical example is provided to demonstrate the relationship between system parameters with the OAMU and the PR.

**Fig 13:**
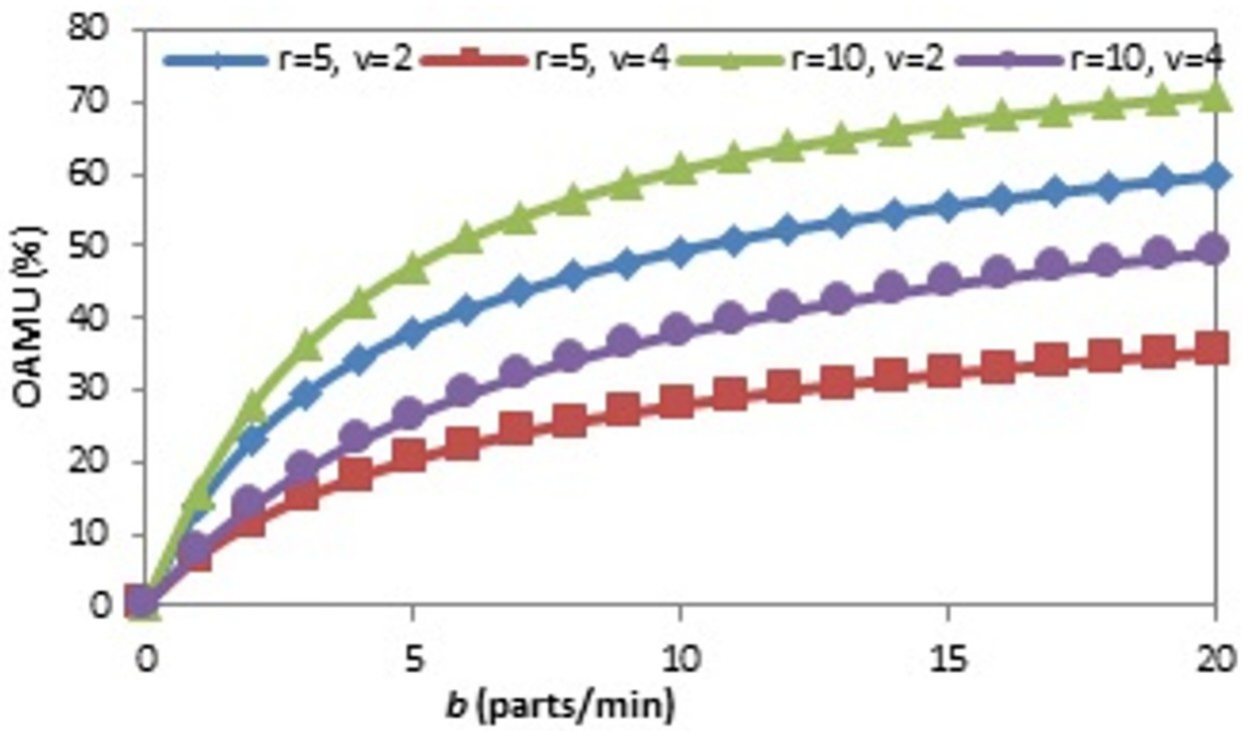
OAMU-b diagram for triple-machine FMC.

**Fig 14:**
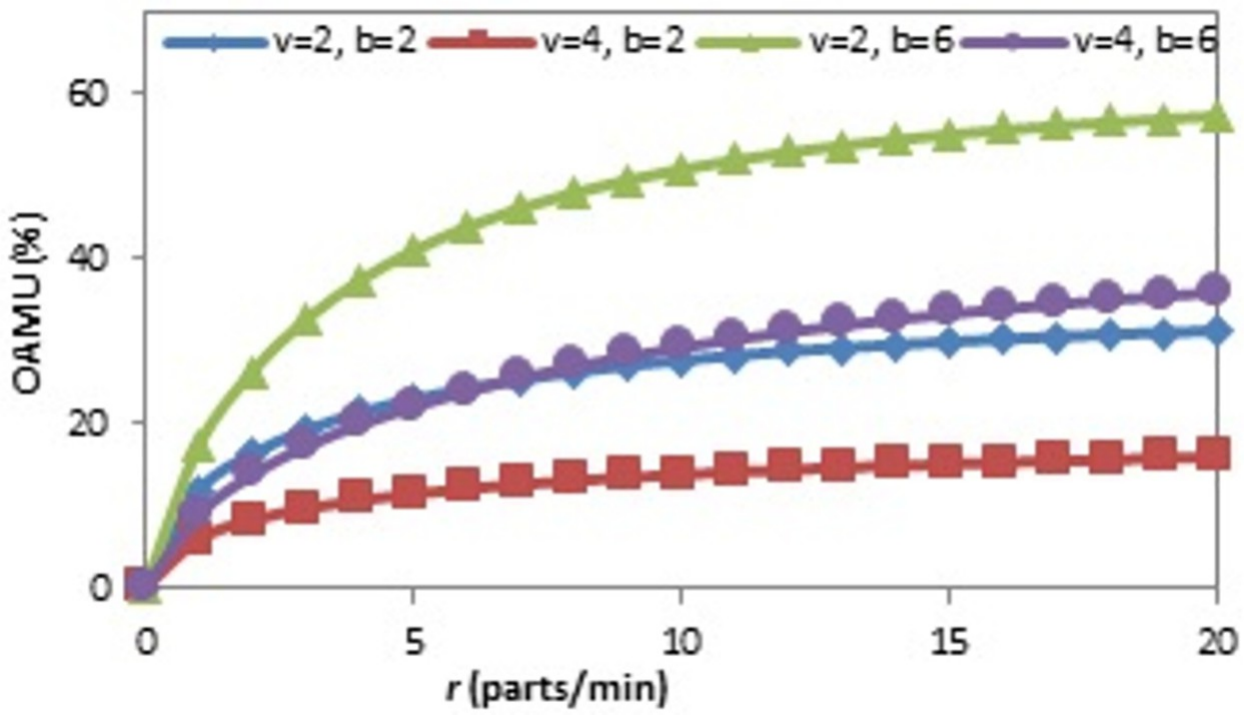
OAMU-r diagram for triple-machine FMC.

**Fig 15:**
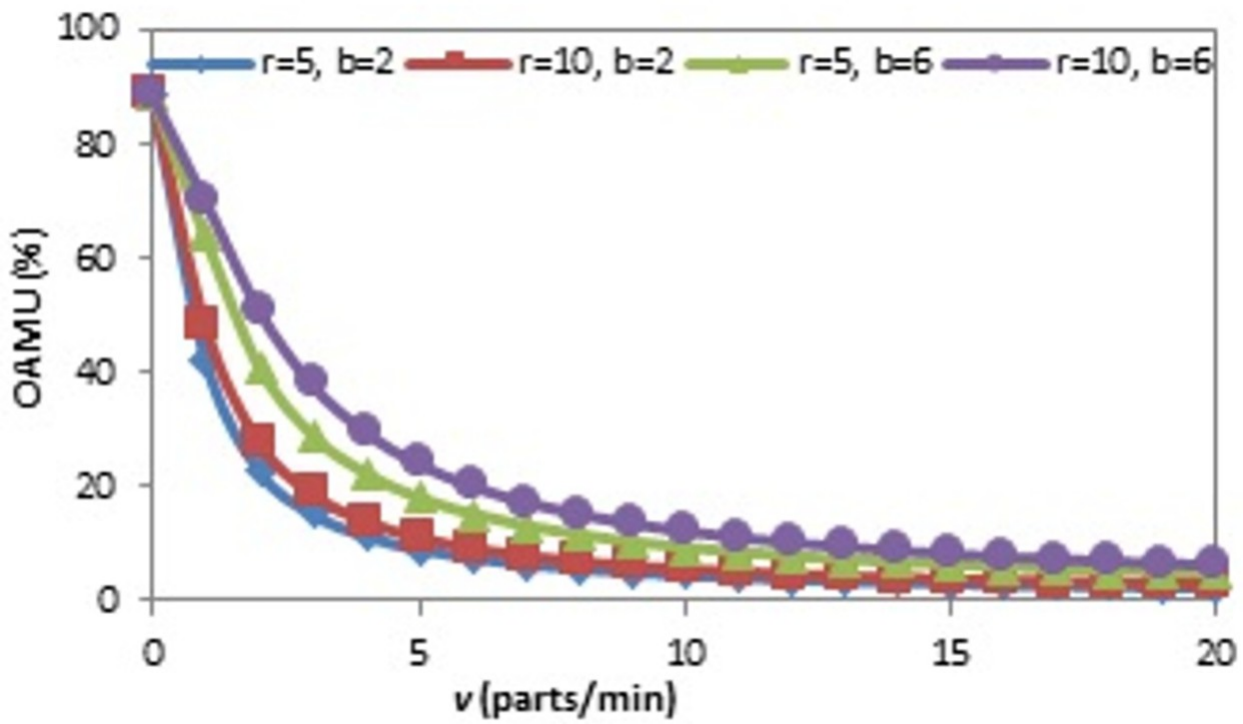
OAMU-v diagram for triple-machine FMC.

Figs 13, 14 and 15 show the effect of (b), (r), and (v) on the OAMU. As illustrated in Fig 13, the OAMU increases as (b) increases. On the other hand, the rate at which the OAMU increases decreases as (b) increases. The effect of (r) on the OAMU is shown in Fig 14. Four combinations of (b) and (v) are considered. The OAMU increases as (v) increases. In addition, the rate at which the OAMU increases decreases as (r) increases. The effect of (v) on the OAMU at various combinations of (b) and (r) rates is shown in Fig 15. The rate at which the OAMU decreases is high at lower (v) values and low at higher (v) values. Generally, the explanation of these relationships is the same as the explanation found in Section 4.1.

**Fig 16:**
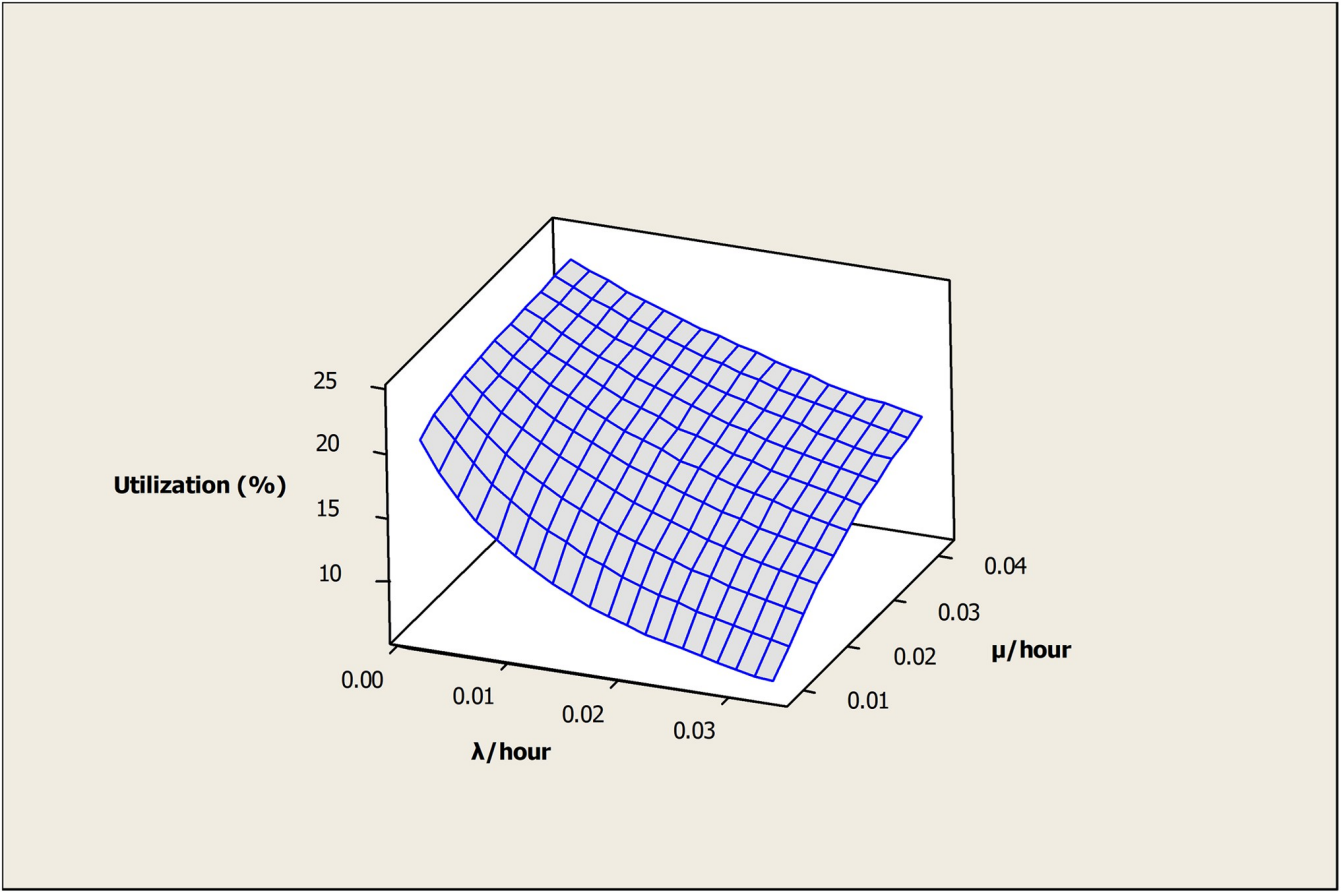
Surface plot of utilization (%) versus μ/hour, λ/hour.

Fig 16 shows the effect of (λ) and (μ) on the OAMU. The OAMU decreases at higher (λ) due to the increase in the downtime, whereas it increases with (μ) due to the decrease in the downtime. Moreover, the OAMU tends to stabilize at higher values of (λ) or (μ). In other words, the OAMU is not sensitive to (λ) at higher (μ) values and it is not sensitive to (μ) at higher (λ) values.

**Fig 17:**
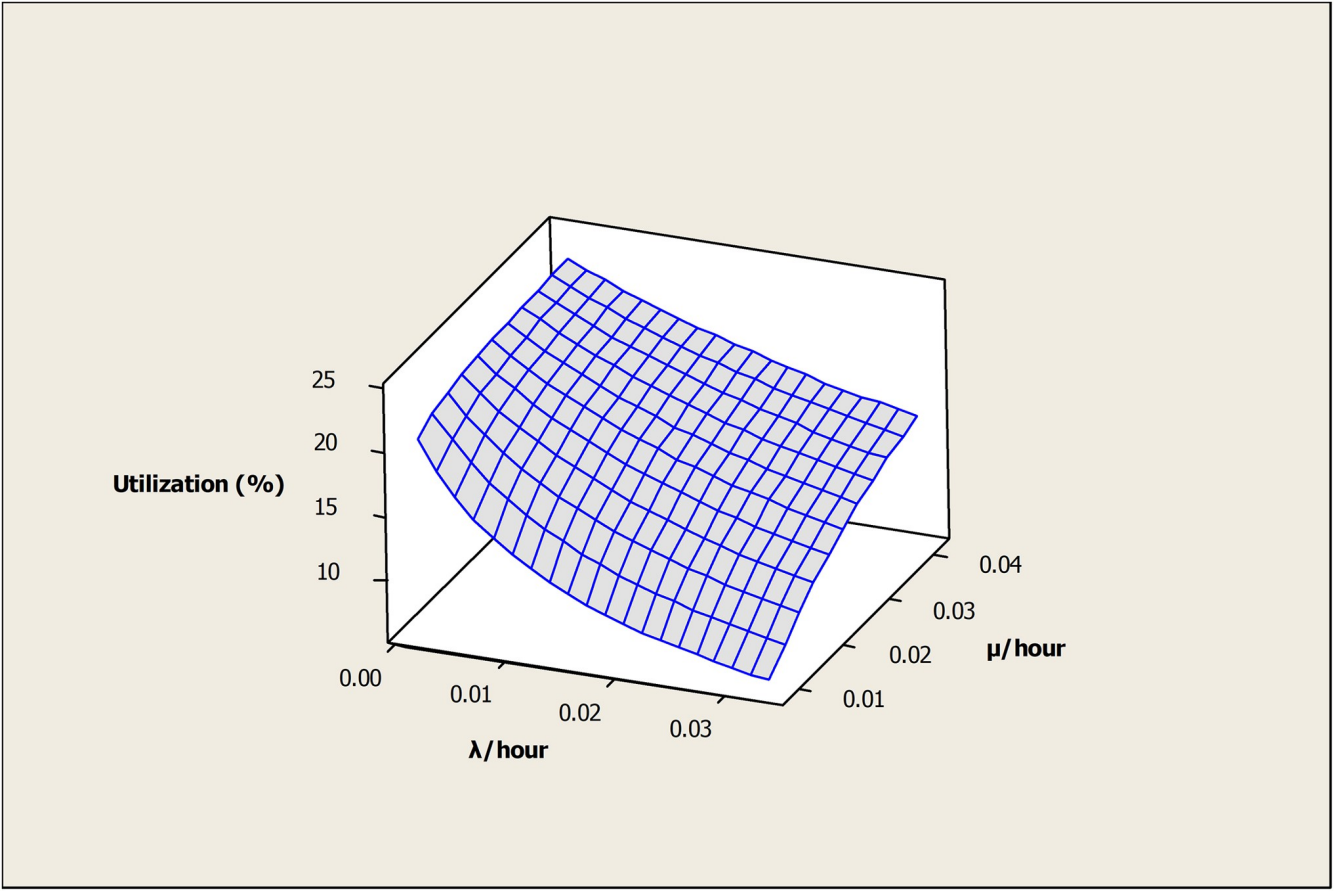
Surface plot of production rate/hour versus μ/hour, λ/hour.

**Fig 18:**
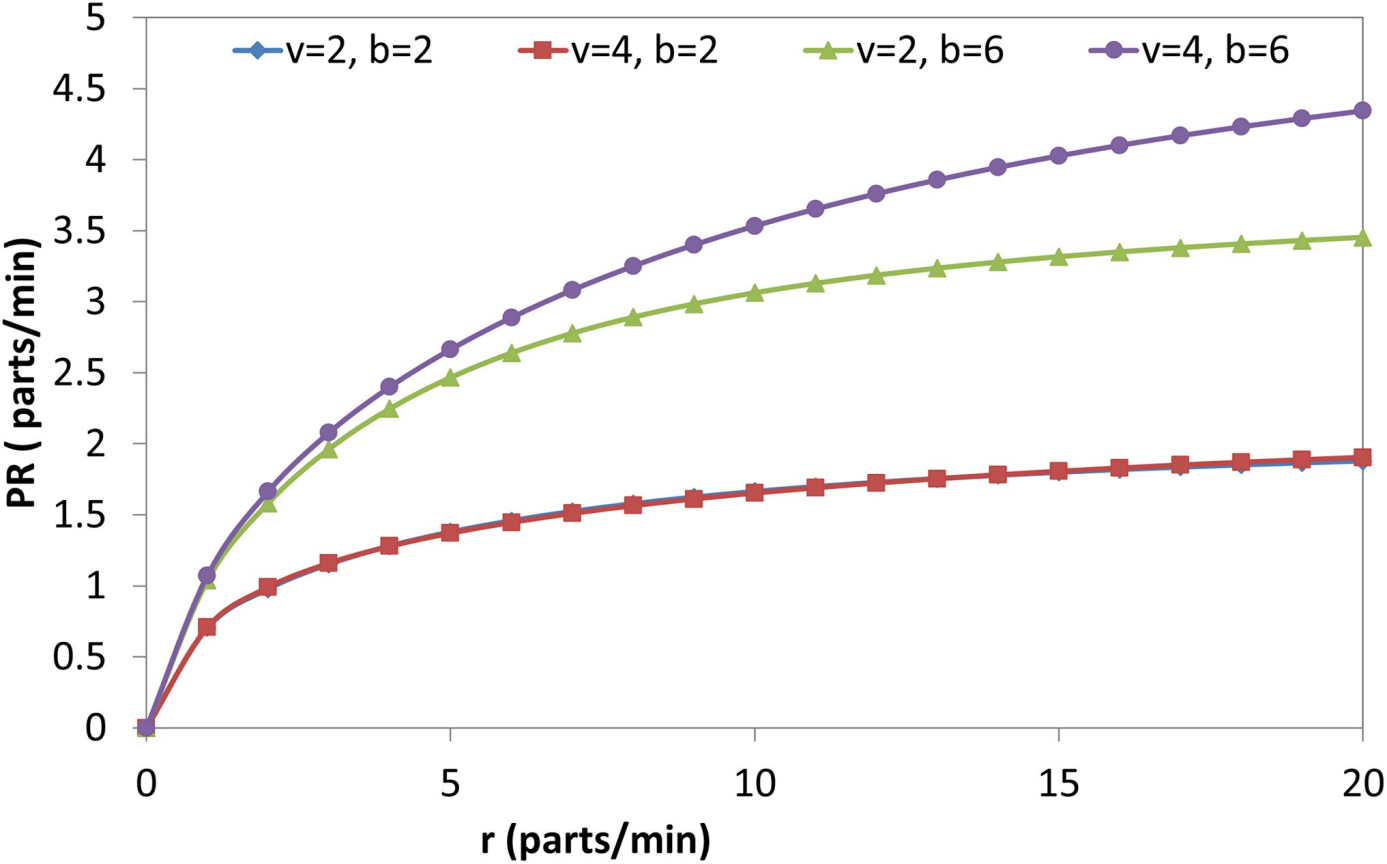
PR-r diagram for triple-machine FMC.

**Fig 19:**
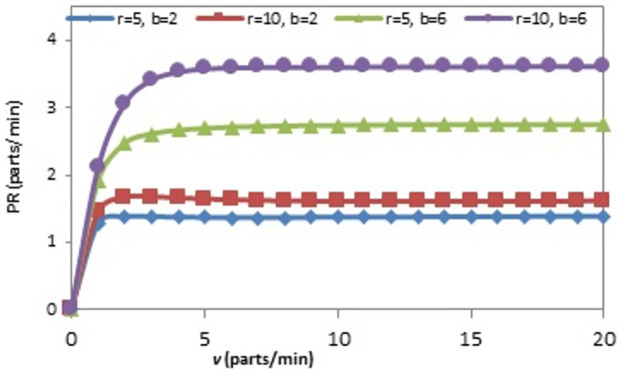
PR-v diagram for triple-machine FMC.

**Fig 20:**
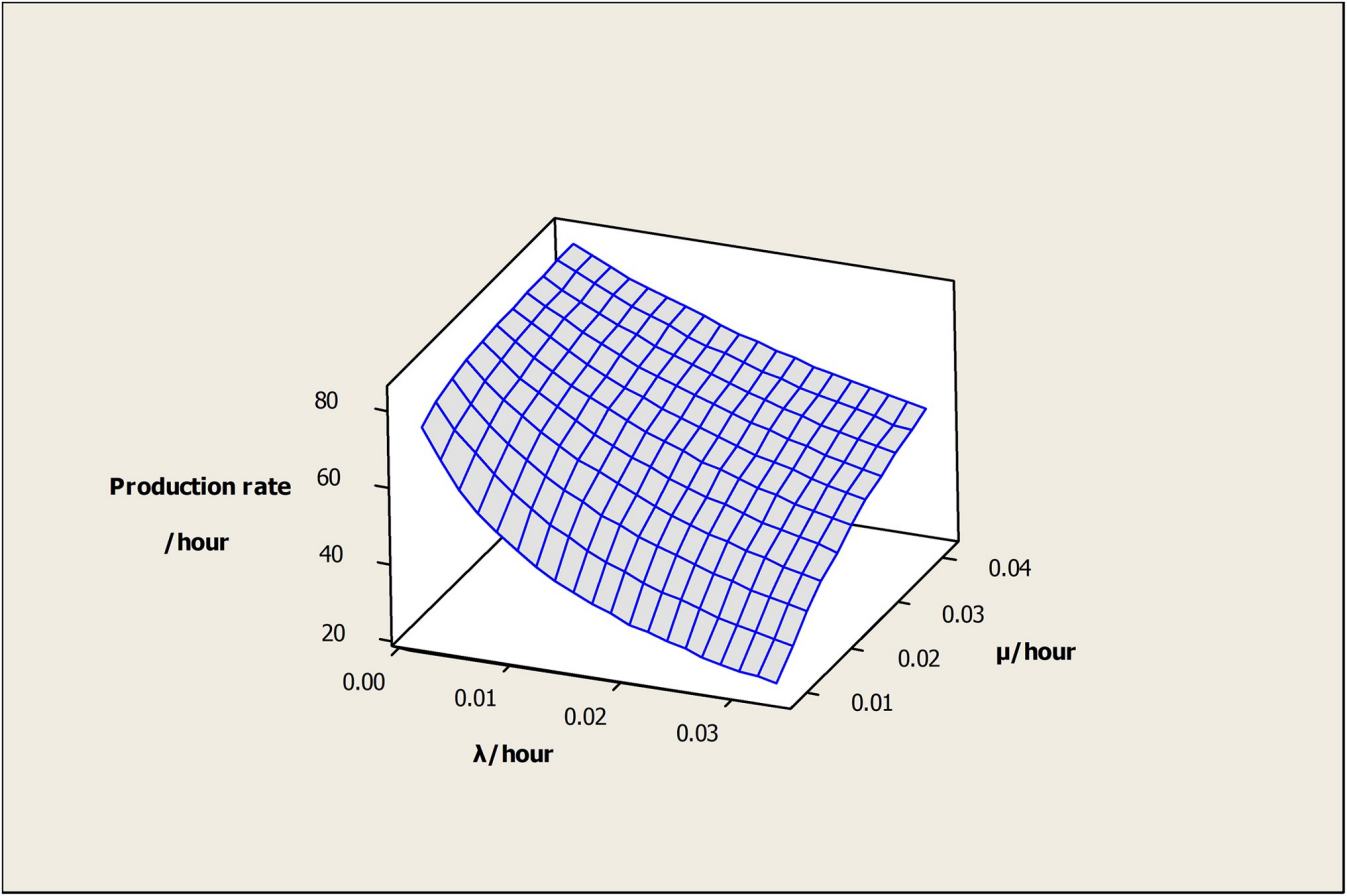
Surface plot of production rate/hour versus μ/hour, λ/hour.

The relationship between the system parameters and the PR for a triple-machine FMC is presented in the next four figures (Figs 17, 18, 19 and 20). The PR increases with the following values: each (b) value, as shown in Fig 17; each (r) value, as shown in Fig 18; each (v) value, as shown in Fig 19; and each (λ) value, as shown in Fig 20. Moreover, it decreases with each (μ) value, as shown in Fig 20. In general, the rate at which the PR increases decreases as each (b), (r), (v), and (λ) value increases, and the rate at which the PR is depressed decreases with each (μ) value. Generally, the explanation of these relationships is the same as the explanation found in Section 4.1.

## 5. Profit optimization of FMC

This section introduces an economic model to assess the net value-added when considering the model constructed in Section 3. The net value-added is defined as the value-added to the products by FMC. The net value-added is represented in the following equation:

$${\text{NP}}_{\text{FMC}}=\left(\text{PR}.\text{VA}-{\text{B}}_{\text{C}}-{\text{R}}_{\text{C}\left(\text{o}\right)}\frac{\text{PR}}{\text{r}}-{\text{R}}_{\text{C}\left(\text{i}\right)}\left(1-\frac{\text{PR}}{\text{r}}\right)-{\text{M}}_{\text{C}\left(\text{o}\right)}\frac{\text{PR}}{\text{v}.\text{n}}-{\text{M}}_{\text{C}\left(\text{i}\right)}\left(1-\frac{\text{PR}}{\text{v}.\text{n}}\right)-\frac{\lambda .\text{REc}}{\mu }\right)\text{T}$$ (13)

The first term in the equation (revenue rate) equals PR×VA×T, where PR is the production rate, VA is the value-added for each part, and T is the production time. T is multiplied by all other terms. Thus, the total cost is divided into three terms: the cost associated with the conveyer belt, the cost associated with the robot, and the cost associated with the machine(s). The conveyer belt cost rate in dollars per unit time is Bc. The cost rate associated with the robot varies depending on its status (i.e., idle vs. working). The cost rate associated with the loading robot is R_C(o)_ multiplied by the percentage of time the robot is working (PR/r). In contrast, the cost rate associated with the idle robot is R_C(i)_ multiplied by the percentage of time the robot is idle (1-PR/r). Similarly, the cost rate associated with the processing machines is M_C(o)_ multiplied by the percentage of the total time that the machines are working (PR/vn). The cost rate associated with the idle machines is M_C(i)_ multiplied by the percentage of the total time the machines are idle (1-PR/vn). The Final Term, λ.REc/μ, represents the cost associated with failure and repairs of the machines.

Consider a single-machine FMC with the following parameters: (VA = $12 /part), (r = 60 parts/hour), (v = 100 parts/hour), (b = 90 parts/hour), (Bc = $6/hour), (R_C(o)_ = $320/hour), (R_C(i)_ = $10/hour), (M_C(o)_ = $500/hour), (M_C(i)_ = $19/hour), (REc = $6/hour), (T = 60 hours), λ = 1, and μ = 2. According to the single FMC model (Section 3), PR = 26.65 parts/hour. Applying the economic model leads to NP_FMC_ = $965/hour.

**Fig 21:**
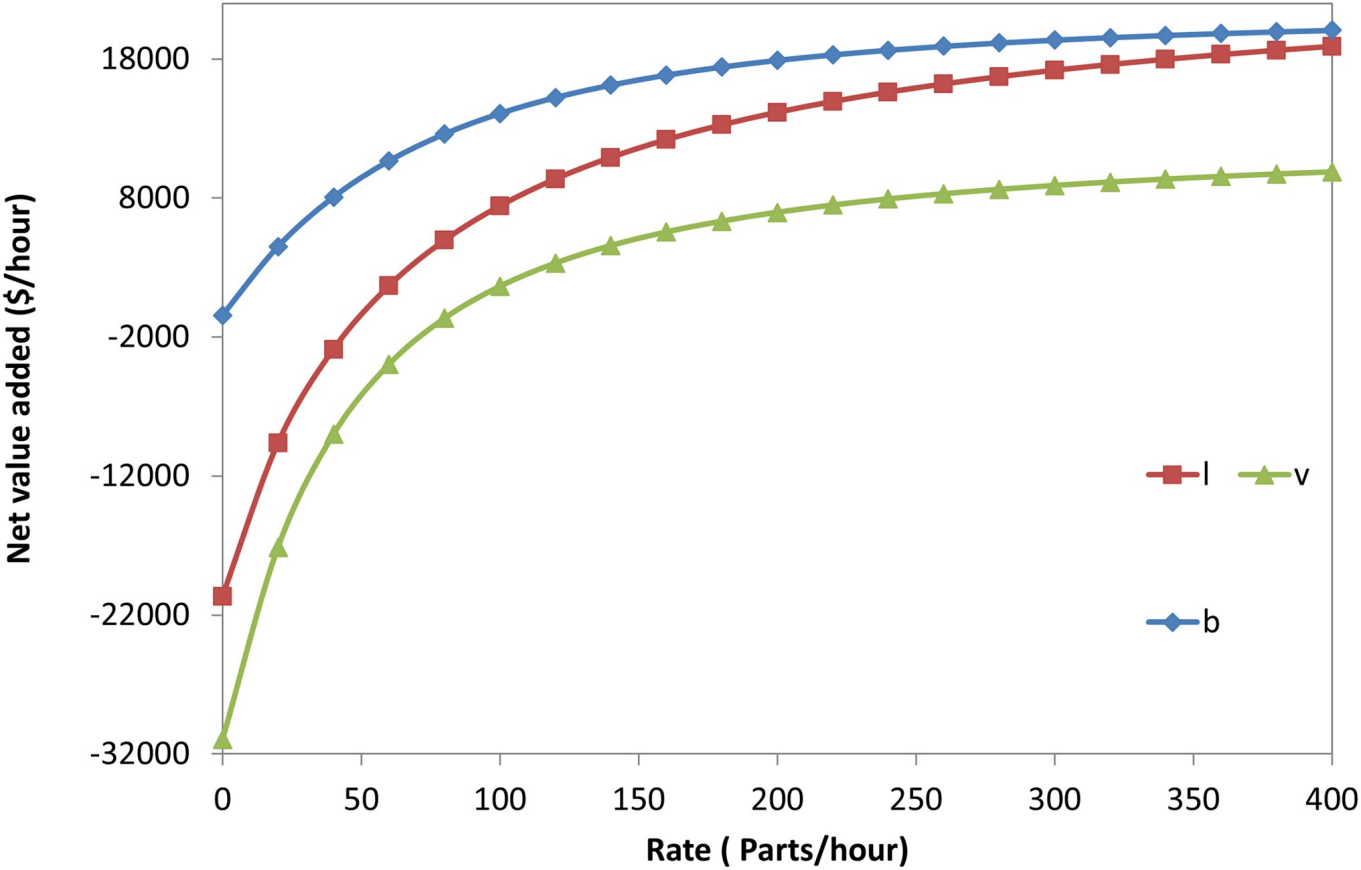
The net value added for a single-machine FMC versus the system parameters.

The net profit increases with conveyer (b), (r), and (v), as shown in Fig 21. The illustrations provided were conducted by varying each parameter against the net profit while the rest were kept constant at the same values as the numerical example. Thus, the shape of the curves is concave; this refers to a sharp increase of the net value at a low rate (value of x-axes) and a mild increase at a higher rate.

Another economic model numerical example was introduced for multi-machine FMC. Consider a triple-machine FMC with the following parameters: (VA = $9/part), (r = 60 parts/hour), (v = 100 parts/min), (b = 90 parts/min), (Bc = $15/hour), (R_C(o)_ = $400/hour), (R_C(i)_ = $12/hour), (M_C(o)_ = $600/hour), (M_C(i)_ = $15/hour), (REc = 6/hour), (T = 60 hours), λ = 1, and μ = 2. According to the multiple-machine FMC model (Section 4), PR = 168.3 parts/hour. Applying the economic model leads to NP_FMC_ = $3,370.6/hour.

**Fig 22:**
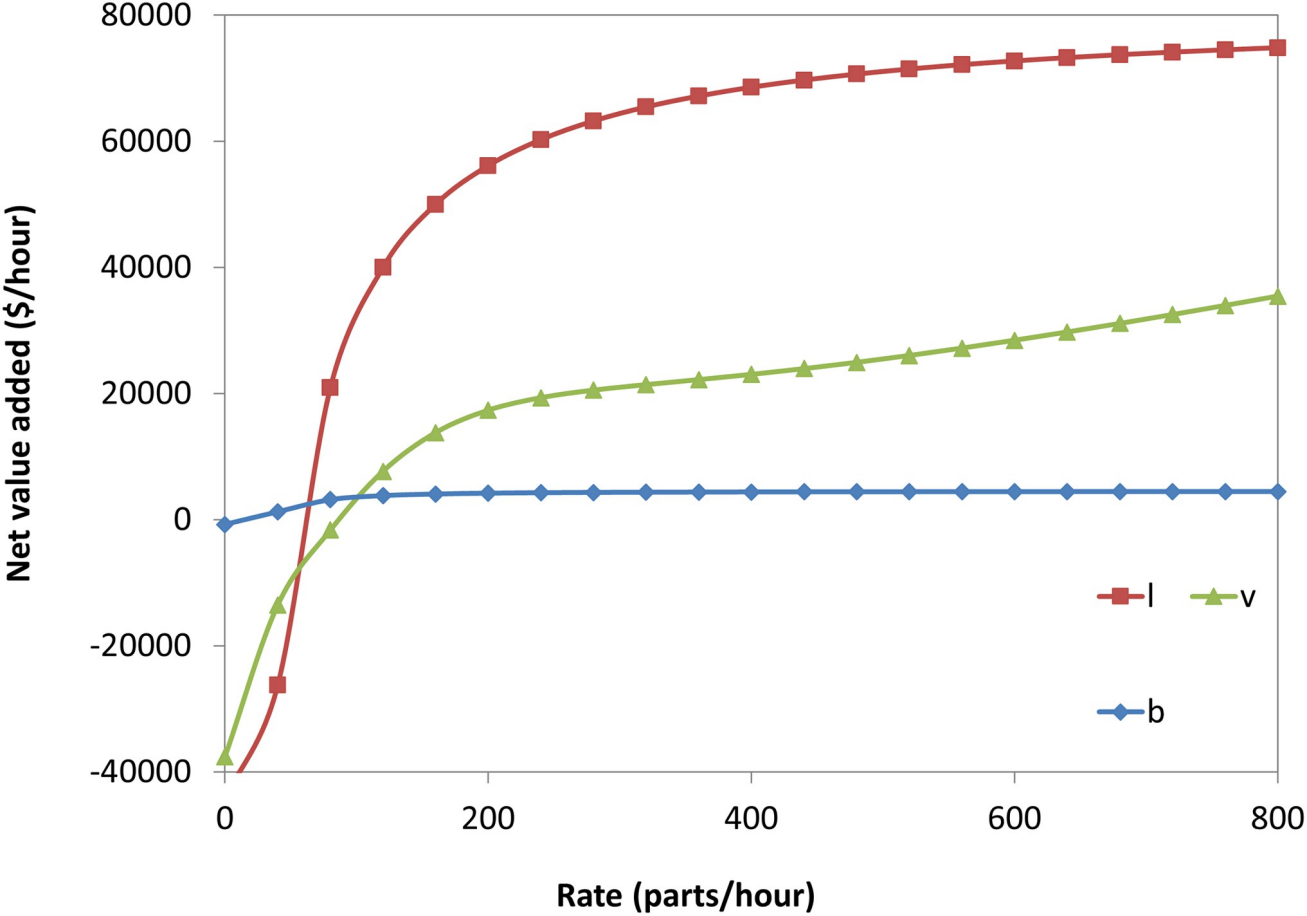
The net value added for a triple-machine FMC versus the system parameters.

As carried out in the single-machine FMC economic model numerical example, the illustrations provided were conducted by varying each parameter against the net profit. At the same time, the rest were kept constant at the same values as the numerical example. As a result, the net value added per unit time increases with (b), (r), and (v) for triple-machine FMC, as shown in Fig 22. The shape of the curves is mostly concave, referring to the decrease of the increasing rate of the net profit at a higher level of all parameters. However, the curves have some additional features and are more advanced than the case of a single machine. Therefore, we conclude that the net profit generally increases with b, r, and v. However, it tends to be stable and maintain a constant value at a high rate.

## 6. Conclusion

The Markov chain is effectively used to model multi-machine FMCs with random distribution parameters. The model can help provide an exact estimation of two FMC performance measures, the OAMU and the PR. Moreover, the model can be used to study the precise trend when changing each parameter of the performance measure; this is very important for industries equipped with FMCs in the design stage. The novelty of this research lies in providing a model to estimate the performance measures and analyze different parameters. For example, the OAMU for single and multi-machine FMC increases with the robot loading, conveyer belt delivery, and machine repairing rates. On the other hand, it decreases with the machine processing and machine-failure rates. However, the increase or decrease is sharp at the low levels of each system parameter (i.e., b, r, v, λ, and μ). Nonetheless, it gradually stabilizes at higher levels. Moreover, the production rate increases with the robot loading, machine processing, and conveyer belt delivery rates. It decreases with the machine-repairing rate. However, the increase or decrease is sharp at the low levels of each system parameter (i.e., b, r, v, λ, and μ). Despite this fact, it gradually stabilizes at higher levels. The further analysis of the economic model shows that the net profit increases with the robot loading rate, the conveyer belt delivery rate, and the machine processing rate. The increase is sharp at the beginning, but it stabilizes at higher levels of all considered measures.

## Supporting information

S1 Appendix Model symbols. (DOCX)
