# Supplementary material for: Markovian analysis of unreliable multi-machine flexible manufacturing cell
Source: PLoS One. 2022 Feb 1;17(2):e0259247. doi: 10.1371/journal.pone.0259247 (PMC8806077; doi:10.1371/journal.pone.0259247)
Supplement: S1 Appendix — (DOCX) [file pone.0259247.s001.docx]

Appendix I. Model Symbols

The model symbols are as follows:

| n | Number of machines |
| --- | --- |
| i | Availability of part to be grasped by the robot (i.e., i = 1 if available and i = 0 if not available) |
| j | Number of machines working on parts (i.e., j = 1, 2, 3, …, n) |
| k | Machine status (i.e., k = 0 if all machines are working and k = 1 if a machine is down) |
| Sijk | Steady state of the FMC in term of parameters i, j, and k. |
| Pijk | Steady-state probability of Sijk |
| r | Robot loading rate to the machine (e.g., parts/hr) |
| v | Machine processing rate (e.g., parts/hr) |
| b | Conveyer belt delivery rate (e.g., parts/hr) |
| λ | Failure rate of the machines (e.g., machines/hr) |
| µ | Machine repairing rate (e.g., machines/hr) |
| NPFMC | Net FMC profit in dollars (net value added onto the product by the FMC) |
| VA | Value-added on each part by the FMC |
| PR | Production rate |
| T | FMC operating time |
| Bc | Cost of operating the conveyer belt in dollars per unit of time |
| Rc(o) | Cost added by the robot during the loading time |
| RC(i) | Cost added by the robot during the idle time in dollars per unit of time |
| Mc(o) | Cost added by the operating machine in dollars per unit of time |
| Mc(i) | Cost added by the idle machine in dollars per unit of time |
| REc | Down machines repairing cost in dollars per unit of time |
